# The Effects of Verbal Encouragement and Compliments on Physical Performance and Psychophysiological Responses During the Repeated Change of Direction Sprint Test

**DOI:** 10.3389/fpsyg.2021.698673

**Published:** 2022-02-18

**Authors:** Hajer Sahli, Monoem Haddad, Nidhal Jebabli, Faten Sahli, Ibrahim Ouergui, Nejmeddine Ouerghi, Nicola Luigi Bragazzi, Makrem Zghibi

**Affiliations:** ^1^High Institute of Sports and Physical Education of Kef, University of Jendouba, El Kef, Tunisia; ^2^Physical Education Department, College of Education, Qatar University, Doha, Qatar; ^3^Higher Institute of Sport and Physical Education of Ksar Said, University of Manouba, Manouba, Tunisia; ^4^Laboratory for Industrial and Applied Mathematics (LIAM), Department of Mathematics and Statistics, York University, Toronto, ON, Canada

**Keywords:** verbal encouragement types, repeated sprint, physical performance, rating of perceived exertion, mood

## Abstract

The general and sports psychology research is limited regarding the difference between the effects of verbal encouragement (VE) or compliment methods during high-intensity functional exercise testing. The purpose of this study was to explore the effects of VE and compliments on the performance of the repeated change-of-direction (RCOD) sprint test. A total of 36 male students in secondary school participated voluntarily in the study. They were divided equally into three homogeneous groups [VE group, compliment group (CG), and control group) and performed a standardized one repetition RCOD. The RCOD (6 × 20 m with 25-s active recovery) test consisted of a 100°change in the direction at every 4 m. Outcomes included performance indices (fast time, average time, and total time), rating of perceived exertion (RPE), and feeling scale scores. VE and the compliment increased the performance indices and RPE compared with the control group. In conclusion, VE during the exercise testing would be more beneficial for optimal performance and RPE compared with the compliment and control groups. However, the moods, during RCOD, reproduce more positively during compliment conditions than the VE and control groups.

## Introduction

Verbal encouragement (VE) is a common method/technique used by coaches and teachers to improve the teaching-learning process (Aguiar et al., 2012; Halouani et al., 2014; Selmi et al., 2017; Belkhiria et al., 2018; Sahli et al., 2020). Theoretically, Wong (2015) defined encouragement as the expression through language to instill courage, perseverance, confidence, inspiration, or hope in a person(s) within the context of addressing a challenging situation or realizing a potential. It is a motivational tool and a procedure to encourage students and athletes to increase their motor tasks and physical fitness performances. The more VE is increased, the more the physical performance increases at high-intensity exercise (Rube and Secher, 1981). The VE can be used to improve the performance of an endurance exercise (Rube and Secher, 1981). Sweeney (2009) considered a significant difference between encouragement and compliment. Encouraging is a source of internal motivation to reach optimal physical performance. Such expressions as “Well done, you are using good strategies,” “you are going to get there,” “I am proud of you,” and “you are capable” are some examples. A compliment is praising and congratulating someone through generating an external motivation to please another person. In terms of compliments, expressions such as “You are capable,” “You are really competent,” “You are too persevering,” and “I appreciate the way you do things” can be used. By complimenting someone, he/she can be more dependent and less reflective than others (Nelsen, 2012). Within the extant literature, the education engagement of students was found to be positively related to teacher care and their encouragements (Wang et al., 2021). In physical education, a teacher who compliments may exercise some kind of power over the student (Nelsen, 2012). However, by encouraging students, they learn more about how to think; they may possibly build their learning strategies while avoiding comparing themselves with each other (Nelsen, 2012).

Brandes and Elvers (2017) reported that the content of the behavior of different coaches could affect the load responses of players. For example, the VE, which includes advice, used by coaches could affect positively the reception and assimilation of feedback in players during training (Cook and Crewther, 2014; Mason et al., 2020; Díaz-García et al., 2021). In addition, previous studies observed that small-sided games with VE increase physical performance with an increase in heart rate, lactate, and rate of perceived exertion (RPE) levels in amateur soccer players (Smith et al., 1977; Rampinini et al., 2007).

Previous studies revealed controversial results regarding the effects of VE on physical exercise. It has been postulated that VE improves athletic performance during exercise (Andreacci et al., 2002; Obmiński and Mroczkowska, 2015). In fact, Andreacci et al. (2002) reported that VE, during a treadmill test, increased peak VO2max and blood lactate concentration. Similarly, other previous studies obtained similar findings and observed the beneficial effects of VE on the performance of athletes compared with subjects without encouragement (Bickers, 1993). In contrast, Obmiński and Mroczkowska (2015) showed that maximal strength performance was not sensitive enough to external verbal stimulation among professional athletes. This corroborates with the study by Argus et al. (2011), who examined the effects of VE on upper-body performance in elite rugby players, confirmed this result. They reported a non-significant small effect of VE on strength performance.

Nevertheless, the VE strategy of the coaches (compliment) could differentially influence exercise performance, indicating the need to separately investigate each verbal strategy. Also, the effect of different VE strategies during short-term exercise as well as RPE and emotional responses of healthy male students in secondary school remains unclear.

In contrast, most sport modalities (e.g., team and individual sports) are characterized by change of direction (COD) as an important factor related to the overall performance (Wong et al., 2012; Dellal and Wong, 2013). In fact, the COD is defined as a preplanned rapid whole-body movement with changes in the velocity or direction (Sheppard et al., 2006). Similarly, Wong et al. (2012) defined repeated change of direction (RCOD) such as a short-duration sprint with COD (10 s), repeated by short rest periods (<30 s).

To the best of our knowledge, no studies have investigated the effects of different types of VE on RCOD performance. Consequently, the main objective of this study was to investigate the effects of VE and compliments on the performance of the repeated change-of-direction (RCOD) sprint test.

## Materials and Methods

### Participants

A total of 36 male students in secondary school (age: 17 ± 0.7 years; body mass: 63 ± 3.6 kg; and height: 168 ± 5.2 cm) participated in this study. The sample size of our study was calculated (G*Power 3.1 software, Germany) with assumed α = 0.05 and an effect size (ES) = 0.2. The results revealed that 33 participants would be needed to reach 80% of statistical power. Therefore, we recruited a few additional participants (*n* = 36) to consider the potential drawing from the study. Students were divided equally into three homogeneous groups: VE group (*n* = 12), compliment group (CG) (*n* = 12), and control group (*n* = 12). There was no significant intergroup difference in age and anthropometric data (i.e., body height, leg length, body mass, and body mass index). Participants regularly participated in physical education classes including ball games, athletics, gymnastics, combat sports, music, and dance (5 h week^–1^). All participants provided written informed consent before participating. None of the participants reported any recent history of hip, knee, or ankle injury. This study was conducted according to the latest version of the Declaration of Helsinki, and the protocol was fully approved by the Ethics Committee of the High Institute of Sport and Physical Education of Kef, the University of Jendouba before the beginning of the tests.

### Procedures

#### Experimental Sessions

The week before the main experiment, RCOD procedure, instruments, and equipment were explained and practiced.

The study consisted of a randomized crossover design in which study participants, of all groups, underwent an RCOD test. Warm-up for RCOD was standardized to 10-min of running, including 3–5 min of light jogging, lateral displacements, dynamic stretching, and jumping. Immediately after the RCOD, the RPE scale (6–20 Borg scale; Borg, 1982) was used to measure the overall physical perceptions of exertion. Also, the feeling scale (FS) was used to measure the affective dimension (pleasure and displeasure), ranging from −5 (very bad) to +5 (very good) (Hardy and Rejeski, 1989). The order of group trials was randomized in order to avoid the possibility of systematic learning effects influencing.

Rating of perceived exertion was collected after the RCOD test using the Borg scale of 6–20 (Borg, 1982). Similarly, FS (Hardy and Rejeski, 1989) was collected after the RCOD test. This scale contained an 11-point single item scale ranging from +5 (very good) to −5 (very bad) with a midpoint of 0 (neutral).

Among the VE group, the following expressions were used: “Go, well done, everything is fine, this is great, don’t give up, great, courage, go ahead, try again, come on, you will get there, I am proud of you, trust yourself, and you can.” However, among the CG, the following expressions were used: “You are capable,” “You are really competent,” “You are too persevering,” and “I appreciate the way you do things.” Those encouraging expression types are frequently used in sport and physical education politics. Given that VE and compliments are both task- and environment-specific (Vallerand, 2004). However, no encouragement was announced to the control group. During the encouragement conditions, the same investigators were present during RCOD trials, and the same level of encouragement was given to participants. The VE or compliment was delivered between sprints (recovery time) to provide more frequent motivation. During the study period, all participants were instructed to maintain their usual physical activity routine. The RCOD test was performed in the same geographical location for the three groups at the same time of the day (±1 h), for all groups, to avoid any potential diurnal variation of performance, and the participants were asked to follow their normal diet during the time of the study.

#### Repeated Change-of-Direction Test

**FIGURE 1:**
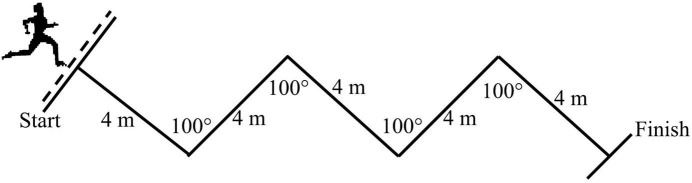
Repeated change of direction test design.

The RCOD (6 × 20 m with 25-s active recovery) test consisted of 100°COD at every 4 m (Figure 1; Beckett et al., 2009). Within each recovery period between sprints, students slowly walked back to the next start point and waited for the auditory signal given by the beeper (Sport Beeper Pro, Best Electronic, France). There was no encouragement between participants during RCOD. The RCOD performance indices were fast time (FT), average time (AT), total time (TT), and fatigue index (FI), as proposed by Glaister et al. (2008). The reliability of the test has been checked using the intraclass coefficient (ICC = 0.946; 95% CI: 0.937–0.951).

### Statistical Analysis

Data were expressed as mean ± SD. The Kolmogorov–Smirnov test was used to assess the normality. One-way ANOVA was used to analyze the differences in the performance indices, RPE, and FS. Bonferroni *post hoc* analyses were used to locate differences among pairs of means when ANOVA revealed significance. ESs for statistical differences were determined. ES was assessed using the following criteria: ≤0.2, trivial; >0.2–0.6, small; >0.6–1.2, moderate; >1.2–2.0, large; and >2.0, very large (10). The level of significance was set at *p* ≤ 0.05. All analyses were carried out using SPSS 16 for Windows (SPSS, version 16 for Windows. Inc., Chicago, IL, United States).

## Results

### Physical Performance

The RCOD performance indices (FT, AT, TT, and FI) were displayed in Table 1. The FT, AT, and TT indices with encouragement and CGs were significantly higher than the control group [*p* < 0.01; ES = (2.34–3.06)]. The AT and TT indices with encouragement were significantly increased than the CG (AT: *p* = 0.028, ES = 1.18; TT: *p* = 0.024, ES = 1.24). However, there was no significant change in FI between the three groups (*p* > 0.05).

**TABLE 1:** Mean values of performance indices for the repeated change of direction test.

	Encouragement group (EC)	Compliment group (CG)	Control group	*P*-value	Effect size
FT (sec)	5.92 ± 0.30^‡^	6.12 ± 0.21^£^	6.51 ± 0.19	0.001**	0.450
AT (sec)	6.14 ± 0.28^**€**^	6.46 ± 0.26^£^	6.88 ± 0.21	0.000***	0.560
TT (sec)	37.03 ± 1.67^**€**^	39.13 ± 1.71^£^	41.66 ± 1.35	0.000***	0.566
FI (%)	4.26 ± 1.63	6.65 ± 4.99	6.56 ± 2.07	0.158	0.142

*Data were expressed as means ± SDs.*

*FT, fast time; AT, average time; TT, total time; FI, fatigue index; ES, effect size.*

**Highly significantly different between the three groups as P < 0.01; ***strictly different between the three groups as P < 0.000; ^‡^significantly different between encouragement vs. control (P < 0.01); ^£^significantly different between compliment vs. control (P < 0.05); ^**€**^significantly different between encouragement vs. compliment (P < 0.05).

### Rating of Perceived Exertion and Feeling Scale

**FIGURE 2:**
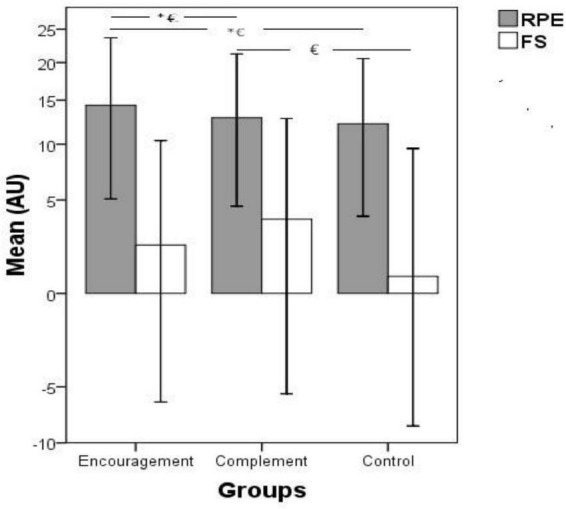
Rate of perceived exertion and feeling scale after the repeated change of direction test. RPE, rate of perceived exertion; FS, feeling scale. *Significant difference between groups on RPE; ^€^significant difference between groups on FS. Data are presented as mean ± SD.

The RPE and FS values recorded after the RCOD are presented in Figure 2. A significant increase in RPE with encouragement group vs. control group (*p* = 0.003; ES = 2.34) was shown, as well as a significant increase in RPE with encouragement group vs. CG (*p* = 0.001; ES = 1.69). However, no significant difference between the CG and control group (*p* = 0.584; ES = 0.80) was observed in RPE. In addition, a significant higher FS score was recorded with the CG when compared with the control group (*p* = 0.000; ES = 3.34). The same difference was also observed between the encouragement group and the control group (*p* = 0.010; ES = 1.71) and between the CG and the encouragement group (*p* = 0.001; ES = 1.78).

## Discussion

This study is the first to investigate the effect of VE and compliments on the performance of the RCOD test. The primary finding of this study was that the use of VE and compliments increased the performance indices (FT, AT, and TT) and RPE compared with the control group. A significant improvement in FS was observed with the CG compared with the encouragement and control groups.

To the best of our knowledge, no studies were interested in examining the effect of verbal compliments on physical performance. Nevertheless, the present results are in line with some previous studies that have shown the beneficial effects of VE on physical performance. Edwards et al. (2018) observed that VE motivated subjects by improving their physical performance during aerobic and sprint exercises. They revealed that VE could improve motivation and stimulate subjects to maintain or increase effort investment during exercise. Similarly, Neto et al. (2015) observed that VE enhanced maximal oxygen uptake, the distances covered, and final heart rate during multistage 20 m shuttle run test. In a recent review, Midgley et al. (2018) showed that VE, every 20 s, increased time to exhaustion during maximal exercise testing more than VE every 60 or 180 s. This study was not specifically designed to encourage participants in regular moments during exercise, but we are concerned about comparing the use of compliment and VE during RCOD. Significant improvements in FT, AT, and TT with VE more than compliment were observed.

As a result, by drawing on the broaden-and-build theory of positive emotions and the significance of applying positive psychology sports research and practice (Wang et al., 2021), we make clear how the positivity of individuals through VE and compliment can result in the physical involvement of the students, the pleasure of learning, physical enjoyment, and well-being while performing physical exercise. The significance of applying positive psychology sports research and practice. Therefore, VE can improve the teaching-learning process, especially for high-intensity exercises (Sahli et al., 2020). Subsequently, potential theoretical and pedagogical implications of external motivation are drawn to enhance the quality and effectiveness of external motivation. Also, encouraging expressions, such as VE, are thought to be a suitable and powered factor not only to induce emotional states but also to subsequently affect performance or cognitive processing (Martín-Loeches et al., 2009). Therefore, it would be required to carefully study the effect of the different characteristics of the encouragement, for instance, the type (encouragement and compliment), expressions, tone, loudness, timing (warm-up, during exercise, and recovery), the language used, frequency of delivery, and the individuality of the participant (age, gender, and trainability). Taken into consideration by our study, these factors were controlled to clearly identify the difference between VE and compliment, physical performance and psychophysiological parameters. These factors support the notion that to optimize maximal test performance, VE should create an adaptive motivational climate during maximal testing by enhancing performance expectancies (competence), supporting autonomy (control), and avoiding controlled or coercive motivation (Midgley et al., 2018).

The results of this study showed a significant increase in VE on RPE during RCOD in comparison with the CG and the control groups. In other words, VE could increase effort investment during RCOD, which is proved by significantly higher maximal RPE. However, no difference was found between the compliment and control conditions. Our results were in line with the study by Andreacci et al. (2002) who observed that RPE significantly increased with VE condition, every 20 s during maximal exercise testing. They suggested that VE does increase effort investment during maximal exercise testing, evidenced by significantly higher maximal RPE. However, the study by Moffatt et al. (1994) showed a reduction in RPE, during submaximal exercise, which delayed the attainment of maximal perception of fatigue with improvement in time to exhaustion of untrained individuals. To the best of our knowledge, no studies have reported the effect of RPE on repeated sprint ability; consequently, it would not be possible to interpret these findings by comparing them with other studies.

This study showed a significant improvement in FS under compliment condition compared with VE and control condition, as well as a significant increase in FS compared with the control condition.

In addition, McCaughtry (2004) insisted on the importance of considering the emotions of students as they learn to improve the teaching-learning process. The emotional factor becomes a determining one in educational learning. Studies conducted in this field consider that the quality of teaching is dependent on the degree of emotions of the students (McCaughtry and Rovegno, 2003; Sutton and Wheatley, 2003; McCaughtry, 2004; Owens and Ennis, 2005; Poulou, 2017). Some recommendations for future studies are suggested. First, the choice of the sample size must be more numerous to improve the statistical power (>90%). Second, in addition to VE, the use of visual feedback could be considered in anaerobic exercise.

## Conclusion

This study investigated the effects of VE and compliments on performance and psychophysiological responses during the RCOD sprint test.

Our data suggest that VE increased the performance of RCOD besides providing the highest values of RPE and FS. In addition, VE can be more beneficial to improve physical performance than compliment. However, the moods, during RCOD, reproduce more positively during the compliment condition than VE.

## Data Availability Statement

The original contributions presented in the study are included in the article/supplementary material, further inquiries can be directed to the corresponding author.

## Ethics Statement

The studies involving human participants were reviewed and approved by the High Institute of Sport and Physical Education of Kef, University of Jendouba. Written informed consent to participate in this study was provided by the participants’ legal guardian/next of kin.

## Author Contributions

HS, MH, NJ, and MZ designed the study and drafted the manuscript. HS and FS performed the experiments. NJ and NO participated in the data analysis. HS, NJ, IO, NB, and MZ revised the critical manuscript. All authors read and approved the final version of the manuscript.

## Conflict of Interest

The authors declare that the research was conducted in the absence of any commercial or financial relationships that could be construed as a potential conflict of interest.

## Publisher’s Note

All claims expressed in this article are solely those of the authors and do not necessarily represent those of their affiliated organizations, or those of the publisher, the editors and the reviewers. Any product that may be evaluated in this article, or claim that may be made by its manufacturer, is not guaranteed or endorsed by the publisher.
